# Leukemia cutis mimicking a sporotrichoid distribution as initial presentation of acute promyelocytic leukemia

**DOI:** 10.1016/j.jdcr.2025.02.044

**Published:** 2025-03-25

**Authors:** Rashi Agrawal, Sara Attari, Margaret S. Newsome, Matthew Powell, Matthew Belcher

**Affiliations:** a Medical College of Georgia at Augusta University, Augusta, Georgia; b Department of Dermatology, Medical College of Georgia at Augusta University, Augusta, Georgia; c Department of Pathology, Medical College of Georgia at Augusta University, Augusta, Georgia

**Keywords:** acute promyelocytic leukemia, leukemia cutis, sporotrichoid

## Background

Leukemia cutis (LC) was first described by Biesiadecki in 1876 as a cutaneous eruption arising from the infiltration of malignant leukocytes into the skin, often reported in association with acute myeloid leukemia (AML) and chronic myeloid leukemia.^1^ The clinical presentation of LC is highly variable, as lesions do not have a pathognomonic morphology or predilection for specific anatomic locations. LC is typically palpable and may appear as papules, nodules, or larger tumors, with rare findings of superficial erosions or deeper ulcerations. The head, trunk, and extremities are all equally affected, and lesions can be solitary, grouped, or disseminated in distribution.^2^ The timing of cutaneous eruption is also inconsistent across patients; onset may occur before, after, or simultaneously along with the diagnosis of leukemia.^3^

Due to this significant variability in clinical presentation, it is pertinent for dermatologists to maintain a high index of suspicion for LC. It is worth mentioning that LC has historically been associated with a poor prognosis, with up to 90% of patients with AML and LC having extramedullary involvement.^4^ Due to this prognostic association, it is important for dermatology providers to familiarize themselves with the widely variable clinical morphologies of LC. Herein, we present a novel presentation of LC skin lesions mimicking a sporotrichoid distribution, a clinical pattern more commonly associated with infectious etiologies.

## Case

A 32-year-old male with no past medical history presented to the emergency department with concerns of weakness, bleeding of his gums, and bruising of his extremities. Associated symptoms also included headache, cough, and dyspnea. Laboratory work-up was significant for hemoglobin of 5.5 g/dL and platelet count of 7000/mm^3^. Complete blood count with differential demonstrated 3% blasts with an angel wing appearance and promyelocytes, findings concerning for acute promyelocytic leukemia (APL).

**Fig 1:**
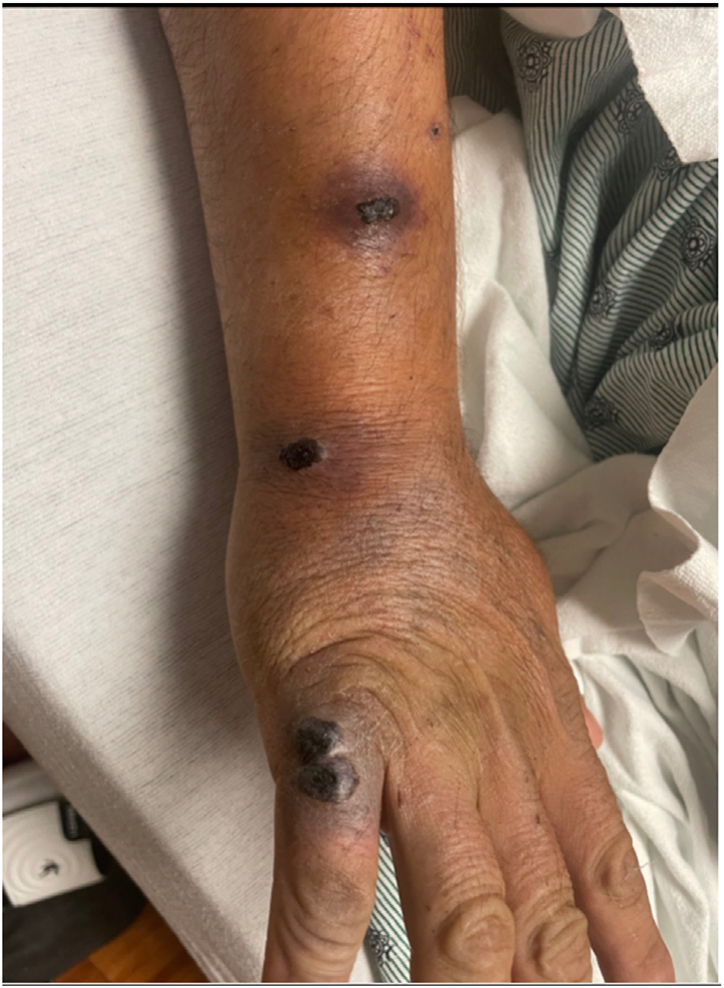
Initial presentation of eschars mimicking a sporotrichoid spread on the patient’s right upper extremity.

Dermatology was consulted for multiple necrotic nodules and eschars in a linear distribution across his right dorsal hand and forearm that had been present for 2 weeks (Fig 1). The patient recently immigrated to the United States from Mexico and had been working on a farm picking blueberries. The cutaneous eruption began as scratches from bush thorns, which subsequently developed into the pictured skin pathology. Although initially painful, lesions were no longer tender at the time of exam. The patient denied any pruritus, bleeding, or other drainage.

**Fig 2:**
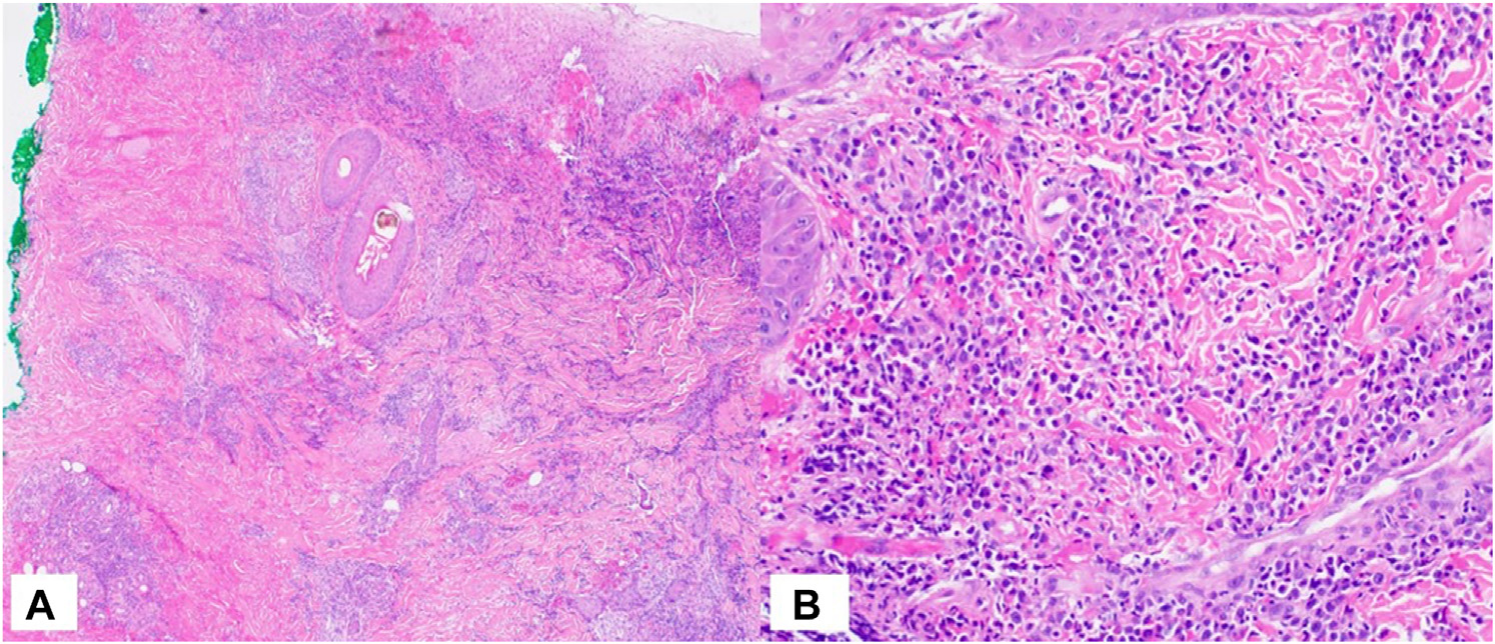
**A,** Punch biopsy demonstrates papillary dermal hemorrhage and edema with underlying interstitial infiltrate at all levels of the biopsy (hematoxylin and eosin, 40×). **B,** The infiltrate throughout the dermis is a mononuclear infiltrate with large, atypical, and hyperchromatic nuclei dispersed between the dermal collagen bundles (hematoxylin and eosin, 200×).

**Fig 3:**
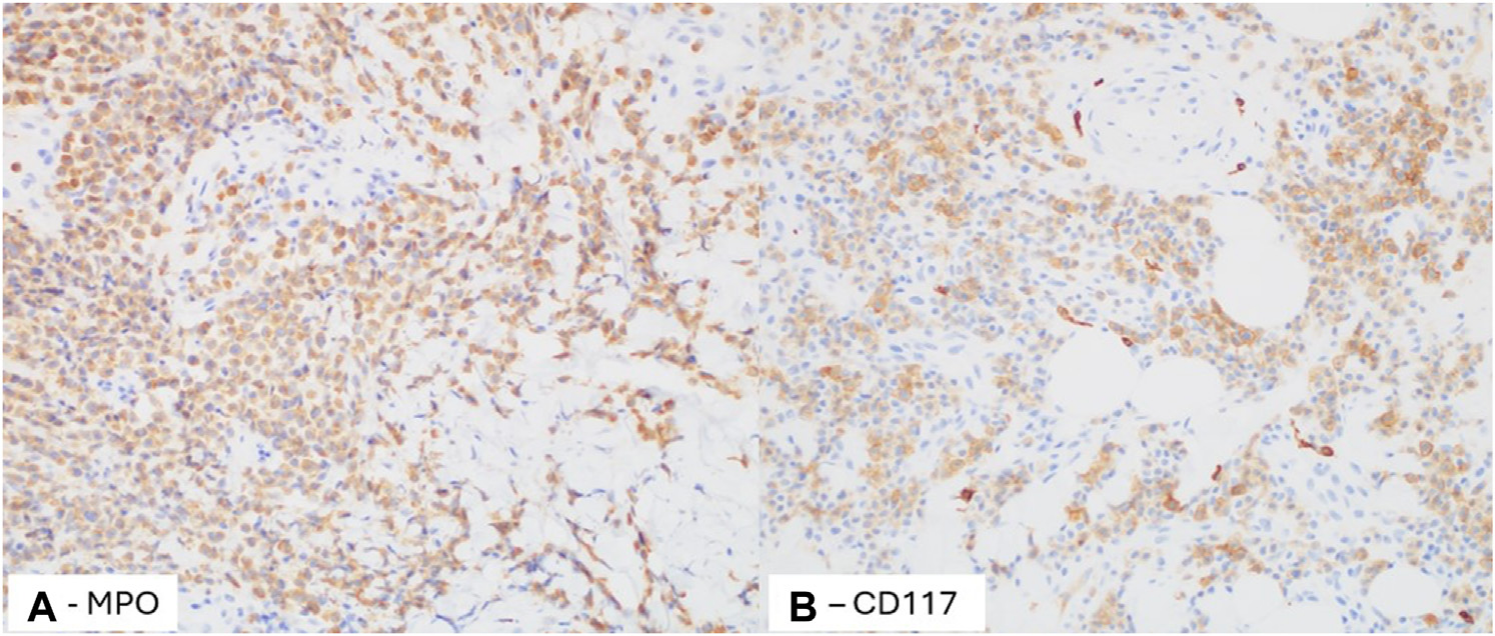
Immunohistochemical evaluation of the mononuclear infiltrate reveals the atypical cells to be diffusely positive for myeloperoxidase (MPO) (**A**) and mostly positive for CD117 (**B**) (200×).

Due to the history of onset after trauma from a plant thorn as well as the sporotrichoid-like distribution of lesions, our differential diagnoses included infectious agents such as sporotrichosis, cat scratch disease, anthrax, leishmaniasis, nocardiosis, tularemia, or atypical mycobacteria. Punch biopsies demonstrated CD117 and myeloperoxidase-positive, large, atypical mononuclear cells (Figs 2 and 3). Grocott's Methenamine silver was negative for fungal organisms, and fungal culture was also negative. This histologic picture was consistent with cutaneous involvement of the patient’s newly diagnosed leukemia (ie, LC).

**Fig 4:**
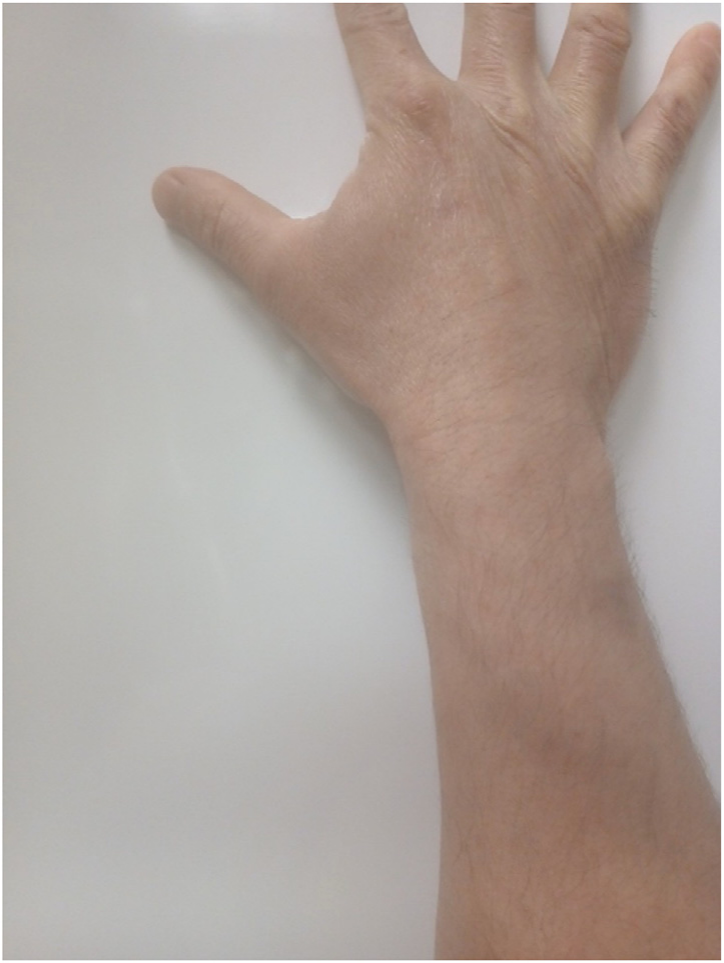
Resolution of skin lesions on patient’s right upper extremity approximately 8 months after initiating treatment for underlying malignancy.

The patient was admitted to the inpatient hematology-oncology team for treatment of his malignancy. He was started on all-trans retinoic acid, hydroxyurea, and cytarabine (later transitioned to arsenic trioxide). The skin lesions were noted to decrease in size within 3 days of induction therapy, fully resolving by 1 month and continuing to be clear approximately 8 months later (Fig 4).

## Discussion

LC is a broad diagnostic term that encompasses any cutaneous manifestation of leukemia. As such, a variety of clinical presentations have been associated with this condition.^5^ In addition to papules, nodules, tumors, and ulcers, there are also reports of more atypical presentations including leonine faces, figurate cutaneous lesions, fingertip hypertrophy, erythema nodosum, guttate psoriasis, chronic paronychia, leukemic vasculitis, and erythroderma.^6^^,^^7^

Our patient presented with black, necrotic-appearing eschars localized to the distal right forearm and hand. The presentation at first appeared to be a site of primary inoculation on the patient’s fifth digit with lesions tracking up the arm reminiscent of a sporotrichoid pattern, defined as linear spread along a path of lymphatic drainage. This distribution is often associated with infectious agents such as sporotrichosis, cat scratch disease, anthrax, leishmaniasis, nocardiosis, tularemia, or atypical mycobacteria.^8^ Intriguingly, sporotrichosis often manifests weeks after traumatic injury by plant thorns and is common in Mexico, which correlated with our patient’s clinical history of blueberry thorn scratches and recent immigration status. However, histopathologic findings ultimately demonstrated cutaneous involvement of the patient’s newly diagnosed APL. As previously mentioned, a diagnosis of LC may precede the diagnosis of leukemia and is a poor prognostic indicator. Therefore, it is important for providers to maintain a high index of suspicion for LC and to be familiar with varying clinical presentations.

Furthermore, LC is a rare manifestation of APL, a distinct type of AML characterized by a t(15;17) chromosomal translocation. In a 2020 systematic review of 184 biopsy-proven, documented instances of LC, only 5 cases were found in APL.^9^ Interestingly, the finding of LC in APL is almost always reported as a sign of relapse, whereas our patient’s cutaneous involvement was a presenting sign of his systemic disease.^10^ Treatment of LC focuses on management of the underlying malignancy: APL is often managed with a combination of all-trans retinoic acid and chemotherapy agents.

In conclusion, our case contributes to the existing literature on atypical presentations of LC. We document a rare finding of LC being one of the primary manifestations of APL and aim to augment the literature by reporting a distinct morphologic distribution mimicking a sporotrichoid distribution. This finding emphasizes the clinical importance of biopsy for atypical lesions, rather than empiric treatment of suspected infections. As LC is associated with a poor prognosis, early diagnosis is a crucial step in management.

## Conflicts of interest

None disclosed.
